# Cases of Fever, with Remarks

**Published:** 1848-11

**Authors:** 

**Affiliations:** Buffalo


ART. IV.—*Cases of Fever, with Remarks.* By the EDITOR.

In this article we purpose to give a few cases of fever, selected from those occurring in practice of which notes have been preserved, and in connection therewith to offer some remarks on the particular points of interest appertaining to their history. The questions involved in the history of the cases, and to which our remarks will have reference, offer scope for much larger discussion than would comport with the limits of a brief article. It is almost unnecessary to premise, that the few comments which time and space will permit us to indulge, will, by no means, embrace such a discussion of these points as their importance and interest claim. What we shall submit, will have, in addition to the little value which they may posses as the results of our own reflections, no other purpose than to serve as suggestives for the reflections of our readers. Our object, in short, is to give in connection with the account of each case, in a brief, discursive manner, some of the ideas which were suggested by the phenomena of the case while it was in progress, and which were of sufficient consequence in our own estimation, to be noted, at the time, in our case book.

*Case* 1.—D. G., aged about thirty; seaman; came under treatment on the 11th of August, 1847. He had on that day left his vessel, which had just arrived at port, having been ill, and confined to his berth, for a week previous. Had taken no remedies save a dose of salts, which had occasioned profuse purging. His symptoms, when first examined, were as follows:—Cephalalgia, occurring in paroxysms of sharp, lancinating pains; skin hot; pulse accelerated, well developed, but not hard. No delirium. Had been purging up to the time of my visit; some cough; respirations accelerated; tongue furred and dry; thirst urgent; no nausea nor vomiting; lassitude, and pains in loins and limbs.

I learned that his habits had been intemperate. One grain of opium was directed to be taken every six hours, which he continued to take for the space of twenty-four hours. The next day, 12th inst., the cephalalgia was relieved, and the diarrhoea arrested. From this date I will give succintly, in anatomical order, a synopsis of the symptoms as presented throughout the disease. Tongue, most of the time, thinly coated, and dry. Sordes collected about the teeth during the latter part of his illness. No vomiting occurred. Diarrhoea did not recur, and the bowels were disposed to costiveness, until the end of the second week, when copious, bloody dejections took place, which occasioned extreme exhaustion. Tenderness over the abdomen was distinct, but not extreme.

A few rose spots (5 or 6) were discovered on the abdomen on the 5th day after my attendance commenced, which were visible for several days, and then disappeared. They were slightly elevated, redness disappearing on pressure. The pulse, most of the time, was but little accelerated. Occasionally febrile exacerbations occurred, but without regularity.

The skin, most of the time, was moist, and, at times, perspiration was profuse.

Toward the end of the second week, mucus dislodged from the posterior nares, was bloody.

Deafness was not present. There was a disposition to somnolency, but there were manifest no indications of mental aberration. Intelligence appeared to be fully preserved through the whole course of the disease, up to a few hours before death. Prostration was considerable, but not extreme, until near the close of the disease, after hemorrhage from the bowels had occurred, when it became very considerable, and was accompanied by subsultus.

Meteorism, so as to occasion visible distension of the abdomen, was not present during the greater part of the disease, but in the latter part became a prominent symptom.

Slight cough existed at first, but, subsequently, this symptom ceased. Perforation of the Intestine occurred on the 13th day after my attendance commenced, and death ensued two days afterward.

*Examination of Abdomen 20 hours after Death.*—Abdomen considerably distended, and unyielding upon pressure, offensive gas escaped on opening peritoneal cavity. Peritoneum presented punctiform redness, like ecchymoses, patches of lymph adherent to mesentery and intestines, and a small quantity of muddy serum, containing flocculi of lymph. Large and small intestines much distended with flatus. Divided the ileum at its connection with the cœcum, and inspected the mucus surface for the space of about two feet above the cœcum. For about a foot, the mucus tissue was uniformly thickened, and softened, and presented several ulcerations, one covering a space of the size of a half dollar. In this large ulcerated patch, a perforation existed of the size of a sixpenny-piece. *Peyer's glands could not be distinguished. It could not be demonstrated that the ulcerations were located in the glandular plates, and the entire membrane appeared to be equally diseased.* The Mesenteric glands were enlarged. Spleen enlarged and softened. The small intestine above the part diseased, contained a pretty consistent yellowish matter. The affected portion did not exhibit this matter, but appeared to be covered with mucus. Owing to the heat of the weather, and want of leisure, other organs were not examined. The record is defective in not showing the appearance of liver and stomach.

*Remarks.*—The first point of inquiry in this case relates to the diagnosis. Was it a case of Typhoid, i. e. common continued fever, or was it Bilious remittent fever? The symptoms of the first few days are not embraced in the history. Whether distinct remissions occurred before the patient came under my charge, I am unable to say. None occurred subsequently. The time of the year was that in which typhoid fever is not common, and in which remittents are frequent. The patient had been exposed to miasmatic influences, and periodic fevers, at that period, were rife among the lake mariners. Remissions after the first few days, are, by no means, uniformally present in cases of remittents, the fevèr lapsing into a continued form. On the other hand, the gastric symptoms, which are so frequently associated with remitting fever, were absent, and several of the symptoms which were present would affiliate the disease to the typhoid genus. The rose spots, (although very few in number) the bloody mucus detached from the Pharynx, and the occurrence of perforation from ulceration of the ileum *par excellence*, are among the characteristic phenomena of the latter. The pathological appearances of the ileum, however, differed from those ordinarily found in well marked cases of Typhoid. The disease was not limited to, nor specially located in the elliptical plates. It does not appear, from the notes, that the elliptical plates above the portion of ileum which was ulcerated, were hypertrophied and softened, as is usually the case. The mesenteric glands were enlarged, and the spleen enlarged and softened, which are among the anatomical characteristics of Typhoid. We are to consider in this inquiry that we have good authority for the statement that ulcerations of the ileum, and well marked disease of Peyer's glands, have been ascertained to have existed in undoubted cases of Remitting fever.

The condition of the intellect was such as rarely obtains in Typhoid fever proceeding to a fatal issue. There were no evidences of that mental aberration constituting what is commonly known as the typhoid delirium. While it is seldom the case that severe typhoid fever of fourteen days duration is wholly unattended by stupor or delirium, it is not uncommon for remittents to run their course, and the intellect remain unaffected.

On reviewing and comparing the facts belonging to the history of this case, we are free to confess our inability to decide, positively, as to its true nosological position, after the arrangement of fevers now generally adopted. Of this difficulty this case is by no means a rare illustration. In a locality like that of Buffalo, where miasmatic and typhoid fevers are now both common, the phenomena of both are not unfrequently intermingled, producing a hybrid form of fever, embracing more or less of the characters of either. We might adduce from our records other cases in demonstration of this fact. There are, in short, in this locality, (and we presume in other localities,) two febrile elements, so to speak—to wit, the periodic, and the typhoid, and the phenomena belonging to both elements may be united in variable proportions.

The inquiry, of late, has been raised, whether the fevers of certain sections where periodic fevers were formerly, and still are, to some extent, pevalent, have not, of late years, undergone a change. We are well assured from our own observations, that this has been the case in this section. The periodic element, year after year, has progressively diminished, and the typhoid element, in like proportion, has developed itself. A few years ago, the fevers of this place, and vicinity, were distinctly periodic, or, to employ the misnomer in common use, "bilious;" now the majority of cases, exclusive of intermittents, are typhoid in their character, or, more properly, common continued. We believe in these views we express the current opinions of the profession of this city.

*Case* 2.—Sept 13, 1847; my attendance was requested upon J. S., aged about 25, a machinist by occupation, a young man of excellent habits, who has resided in this city for several years, and has always enjoyed vigorous health.

The history of the case up to the time of my attendance is, briefly, as follows: He was taken ill on the 2d inst. (ten days.) Previously to that date he had been ailing for several days, complaining chiefly of head-ache. On the 2d, he had a chill, and took to his bed. Since then, up to the time of my attendance, he was under the care of a homœopathic practitioner. His symptoms before I saw him, so far as I could ascertain respecting them, denoted continued fever from the first. Headache was the most prominent symptom for the first few days. His intellect appeared to be clear for the first few days, but he has since been constantly wandering. On one occasion he got out of bed, and went into the adjoining room, with the design of going out of doors. With this exception the delirium has been of a quiet, muttering character. He has had no diarrhoea. Bowels have been soluble. Has had no epistaxis. Had slight nausea and vomiting on one occasion.

The present symptoms are as follows: Mind apparently collected when the patient is roused; answers questions coherently, but relapses into a state of somnolency directly after being addressed. Lies on the back with lower jaw depressed. Tongue thickly coated, dry, and hard towards the tip. Teeth and lips incrusted with sordes. Some deafness. Skin cool. Pulse 100. No meteorism. Abdomen and chest exhibit a small dusky eruption, redness disappearing on pressure. Obscure tenderness, and gurgling on pressure over right iliac region. Makes no complaint. Bowels moved last evening. The medicines hitherto given produced no appreciable results; their nature not known. *The only diet allowed has been rice-water, until to-day, when a little arrow root has been given.*

Directed, for treatment, essence of beef, and a little brandy and water.

Sept. 18. Fever continues, but symptoms have manifestly improved. He has less muttering delirium, and less sordes. Tongue is moist. Pulse more developed, and less frequent. The pulse, for a day or two after the last record, was very feeble and intermitting. The bowels are soluble, moving once or twice daily, discharges being rather thin, and of a yellowish color. There has been no cough. The eruption has continued to be visible. The treatment has consisted of essence of beef, and milk porridge, given hourly, in alternation. Brandy hourly. S. Quiniæ gr. i., every four hours.

20th. Substituted yesterday maderia wine for the brandy, and allowed a little peach, and water-mellon. He has had, of late, until to-day, two exacerbations during the daytime. To day there has been more delirium, and the tongue is dry. No distension of abdomen; obscure tenderness continues. Three dejections since last evening.

Oct. 12. This patient is now convalescent. He became fairly so on, or about the 28th day after first taking to his bed. Convalescence was established by a gradual amelioration of symptoms. For several days preceding his convalescence, he sweat profusely at night. Delirium continued for several days after he could be pronounced convalescent. He imagined he had acquired great wealth—owned steamboats, horses, carriages, etc., and that he had large quantities of gold coin on hand. These delusions have now entirely disappeared, and he is rapidly regaining strength.

This patient has continued to enjoy good health up to the present time.

*Remarks.*—This case, the details of which, although rather sparingly recorded, are fresh in my recollection, illustrates some important points connected with the therapeutics of fever. As regards diagnosis, the case was doubtless one of continued fever, but whether it was properly to be considered typhus, or typhoid, may admit of discussion. We will not, however, stop to discuss that question, passing it by with the single remark, that to define the boundary lines between these two genera, or species, (whichever they may be considered) is certainly not an easier task than to determine, abstractedly, the dividing line which separates the animal from the vegetable creation.

The case may be considered, in the first place, as exemplifying the career of continued fever of a grave character without medicinal treatment. Quinine, in dose of gr. 1, every four hours, during a portion of the disease, constituted the sole medicinal remedy employed after the case came under my care; and there are good grounds for believing, that, in so far as the case was treated by a homœopathist, the treatment was really homœopathic. This statement is quite necessary, inasmuch as a vast deal of active medication is practiced under the fraudulent guise of homœopathy. That Quinine exerts little or no power either to shorten, or modify the course of continued fever, I have satisfied myself by repeated experimental observations. It was prescribed in this case because it was a remedy as nugatory as any entitled to be considered remedial, and simply, because I was unwilling that it should be said, provided the case terminated fatally, that nothing was given in the form of medicine. This course was not pursued for the sake of experiment, but in accordance with the golden rule of therapeutics, *not to prescribe active medicinal agents, except with a view to some definite purpose which we have reason to believe may be safely fulfilled, and, if fulfilled, will place the patient in a state more favorable for recovery.* We beg leave to commend this maxim to the consideration of our readers. Guided by it, many cases of fever, of even a grave type, will call for very little medication.

In the second place, this case illustrates the importance of alimentation in fever, The patient for the first ten days had taken only rice-water for diet. I found him greatly prostrated, and rapidly sinking. Under the influence of stimulants, and nutriment, he speedily improved; and although his disease was protracted to the 28th day, he passed through it without accident, or any untoward symptoms. Patients in the state in which this young man was, rarely ask for food, or even drink. The mental condition is such that they are insensible to natural instincts, and their senses are dormant. The judgment of the physician and attendants must supply the place of both instinct and sensation. In other words, nutritious ingesta must be administered without reference to the desires, or even taste of the patient. That assimilation is capable of taking place in a limited degree, notwithstanding the febrile career, was apparent in this case, from the fact that the patient perceptibly gained strength while the fever was in progress. A few days after my attendance commenced, he was able to change his position in bed; to raise himself on his elbow to receive food and drinks, and to assist himself in passing his evacuations; and, in the mean time, he improved in other respects. We are reminded of the significant language of Dr. Graves on this topic: "If," says he, "I have had more success than some others in the treatment of fever, it is owing to my having followed the advice of a shrewd country practitioner, who advised me not to let my patients die of starvation!" (I give the quotation in substance, not verbatim.) That patients with fever have been permitted to die from inanition, seems to us but too probable. The diet suitable for fever cases is milk porridge, and the juice of meat; and by the latter is not meant the beef tea as usually prepared, which is, in fact, hot water flavored with animal *osmazome,* but the pure concentrated essence, obtained by boiling a sufficient quantity of meat in a vessel placed in boiling water, with the addition of very little, if any, water to the meat. The practitioner will find it necessarry to attend to these details, if he wishes to secure for his patient the advantages of proper alimentation.

The continuance of delirium for some days after convalescence was distinctly pronounced, is an unusual circumstance. Its character, in this case, was that of exaltation in sentiments and ideas, and it may be perhaps supposed that it proceeded from the exhilerating influences of returning health. The patient, while it lasted, imagined himself possessed of the most ample means for temporal enjoyment. The delusion disappeared suddenly. The patient said that he thought the matter all over one morning, and came to the conclusion that his circumstances were the same as before his illness.

The mental condition in continued fever, during its progress, is not always fully appreciated by medical, and other attendants, when the patient does not give evidence of wandering by incoherent conversation. Patients frequently reply to questions coherently, and with apparent rationality, by a kind of habitual action of the mind, when no dependence is to be placed on the correctness of their replies. In the case just given, for example, while the bowels were daily open, I enquired, on one occasion, when he had the last movement, and his reply was that his bowels had not moved for two weeks! I had supposed that this patient was partially rational through the febrile career, but he informed me, after his recovery, that from the day he took to his bed, up to his convalescence, the period was almost an utter blank; he had an indistinct recollection of but a few striking events. He did not remember that I had visited him before his amendment occurred. In another case, of a medical friend, who passed through what appeared to be a very mild form of Typhoid fever, without exhibiting any indications of mental aberration, he stated, after his recovery, that he was able to recal but few events of his illness. A practical consideration connected with these facts is, to avoid placing too much reliance on the information derived by inquiries addressed to patients during the progress of Continued fever, even when they appear to have a rational command of their intellectual faculties.

*Case* 3.—D M., aged 20; mariner. Habits good. Entered the *Buffalo Hospital of the Sisters of Charity*, August 23d, 1848.

Precise period of illness, and the early history of the case, unknown. Had been ill several days on board vessel, from which he was transferred to hospital. Arrived at this port on the day of his entrance. Delirium manifest by incoherent conversation. Face presents a dingy color. Surface of body generally, exhibits congestion. Eyes suffused. Expression stolid, somewhat like one intoxicated. Skin bathed in profuse perspiration. Pulse very small, and irregular. Tongue covered with dark coating, dry and hard in centre. Sordes on teeth. Complains of nothing; says he feels very well. Abdomen not distended, and no appreciable tenderness, nor gurgling. Dejections frequent, characters not ascertained. Is somnolent, and not easily roused. Treatment: Carb. Ammoniæ, grs. v. every two hours; two drachms of brandy every two hours. Lemonade.

24th. Perspiration diminished. Pulse more developed. Other symptoms the same. Treatment continued, with milk porridge for diet. Surface of abdomen mottled with faint red maculae, as if eruption was about to appear.

25th. Aspect improved. Pulse well developed, and but moderately accelerated. Skin warm and mellow, still congested. Mind more clear. Tongue moist. Treatment continued.

P. M. Tongue dry and hard. Pulse 100. Several dejections. Eruption not more developed. Skin moist. Continues somnolent, but is easily roused. Treatment continued.

26th. Copious, but faint eruption over abdomen and chest, extending, also, over upper and lower extremities. Gurgling in abdomen on pressure. Pulse 96. Skin cool and moist. No dejections. No distension of abdomen, nor tenderness on pressure.

P. M. No material alteration in symptoms. Treatment continued.

27th. Tongue moist. Two dejections during night. General aspect better. Eruption more distinct, extending even to feet and hands. *Redness does not disappear upon pressure; maculae not elevated, color somewhat livid.* Congested state of skin continues: presents an appearance as if the surface had been exposed to intense cold, consisting in a diffused, livid redness, which disappears momentarily on pressure. Skin dry and of natural temperature. Pulse 80, and feeble. No distension of abdomen. No evidences of delirium. No disposition to sleep during visit. Appears indifferent to persons and things around him. Treatment: Pulv. Camphoræ, grs. ii every two hours. Some deafness has existed throughout the disease.

28th. Aspect better. Tongue moist, and sides cleaning. Congested state of skin has greatly subsided. Eruption much faded. One dejection during night. Got up without assistance to relieve the bowels and bladder. Smiles when asked if he would relish a beefsteak. Pulse 80. Skin feels natural. Treatment continued with addition of essence of beef to diet. Sweat profusely last night. He now changes his position in bed without aid, and frequently lies upon his side. Treatment continued.

29th. Tongue less coated, moist. Sordes still collects about teeth. No dejection. Maculae have disappeared from abdomen; still visible, but faint, upon extremities. Abdomen undistended. Arises without assistance to urinate. Pulse 72. Treatment continued.

30th. Countenance much improved. Appearance of tongue better. No dejection. Pulse 56. Treatment continued.

31st. Improving. Got up, of his own accord, and dressed, but felt compelled to lie down immediately. No dejection. Deafness continues. Ol Ricini ℥ss.

Sept. 1st. One dejection from oil. Continues improving. Treatment: P. Camp. grs. ii every four hours.

Sept 2nd. Sitting up. Deafness continues. No medicine.

This patient continued to improve without any bad symptoms No medicine was afterwards prescribed, and he was discharged well, on the 12th inst.

*Remarks.*—The point to which we would direct attention in the foregoing case, in connection with the general symptoms denoting gravity, is the course of treatment as respects medicine and diet. The case was one of well marked Typhus, and the symptoms, at the entrance of the patient, led to the apprehension that the disease might speedily run on to a fatal issue. The only therapeutical indication present, however, was to sustain the vital forces by stimulants conjoined with nutriment. The history shows that speedily, under this plan, the symptoms assumed a favorable character. Afterward the only treatment pursued consisted in the regular administration of camphor in small doses, brandy, and nutriment, with the exception of a single dose of castor oil after convalescence was declared. The reader will please notice how much the strength of the patient was preserved, being able to rise, without assistance, to relieve the bowels, &c., even before convalescence was distinctly pronounced, showing, as we think, that muscular strength may be actually, in some degree, regained, during the progress of fever, under the use of stimulant remedies and alimentation, those therapeutical measures being at the same time avoided, which would be likely to compromise the vital forces.

*Case* 4.—A German, aged about 25, was enlisted at the U. S. Recruiting Rendevous on the 12th Nov., 1847. He appeared at that time perfectly well and robust. On the 13th and 14th inst. it was noticed that he appeared dull, and did not seem to be well. On the 15th, A. M., my attendance was requested. As he spoke no English, my communication with him was conducted through an interpreter, and was somewhat imperfect. The temperature of the skin was elevated; pulse accelerated, and neither full nor hard; hands tremulous. Bowels open. Complained of no pain, but said he felt fatigued. I was informed that he manifested delirium during the night. The symptoms led me to suspect delirium tremens, but I could not ascertain that he had been indulging in stimulants, and his appearance had denoted that he was temperate in his habits. He had been in the country for four months. I prescribed as follows;—℞. Prot. Chlor Hydrarg. grs. x, Pulv. Rhei. grs. x. At evening I was unable to learn whether cathartic operation had been free, nor to obtain any information from the patient, the interpreter not being at hand. The prescription for this evening was, P. Opii gr. i, to be repeated in four hours if he did not sleep. Up to this time I had made no diagnosis, and was under the impression that the disease might be due to intemperance.

16th. Patient had been more quiet through the night, but delirious He was sitting up, and dressed, but appearing greatly prostrated. Pulse very rapid. Appearance of tongue not morbid, but tremulous, and not readily protruded. Says he has no pain. ℞. P. Doveri grs. iv, every six hours. At evening, aspect worse. Respiration accelerated. Pulse very rapid. Skin has a dry, scorching heat. Constant wandering of mind; answers questions coherently, but apparently after taking a long time for deliberation. Mutters, and frequently attempts to get out of bed. Did get up on one occasion when unobserved, and went to the sink to relieve the bowels. No desire for food. Asks for nothing. Has taken, to-day, milk porridge by direction. Countenance has a sunken aspect, and an expression of vacancy, or astonishment. Coughs occasionally. No meteorism of abdomen, nor evidences of abdominal tenderness. A few small rose spots perceptible on the abdomen.

On this day the symptoms declared the disease to be typhus, resembling some of the cases of the so-called ship fever, which, at that time, prevailed among immigrants arriving at this place.

17th. Greater prostration. Pulse 120. Respiration labored and suspirious. Tongue, teeth, and lips loaded with sordes, which has accumulated since yesterday. Constant somnolency; mutters frequently in sleep. Coughs occasionally, and expectoration is sanguinolent. Two or three dejections have occurred, which were liquid and yellow. No meteorism; firm pressure on abdomen causes groaning. Eruption indistinct. Surface of body has less heat. Has much difficulty in protruding the tongue. Asks for nothing. Countenance hippocratic. Treatment: Liquor. Acet. Ammon. ℥ss. every two hours; ale hourly; milk porridge. At evening symptoms worse. Pulse as frequent, and more feeble. Skin cool. Respiration shorter and suspirious. Substituted brandy for the ale. Porridge and ammonia continued.

18. Died at 2, A. M. Got out of bed, and walked a few steps a few moments before his death. Autopsy not practicable.

*Remarks.* The history of this case illustrates the rapidly fatal progress of fever occurring in a subject, to all appearance, in perfect health the day before he was attacked with the disease, and proceeding to its fatal issue, not in consequence of the intensity of local complications, but purely from the intensity of the febrile affection. What are we to understand by the febrile affection; in other words, what are the pathological conditions which this expression involves? This inquiry leads us, at once, to the ætiology, and essential nature of continued fever, subjects which have been discussed for centuries, and are still open for discussion. We will only remark, that, to our own mind, the history of such cases, reasoning *par voie d'exclusion*, and by analogy, carries in a forcible manner the impression of a poisonous agency, which, affecting primarily the circulating fluid—the blood—occasions universal disorder, and destroys by its influences upon the sources of vital activity.

*Case* 5.—On the 5th Sept., 1848, my attendance was requested upon Mr. C., aged about 21, of excellent habits, and who had enjoyed good health. He had a few weeks before his illness made a journey westward, and had lately recovered from Pertussis. His duties, as a clerk, had been onerous, and he had had, for some weeks, an appearance of impaired health. For several days before the attack he had complained of feeling unwell. I saw him first on the evening of the 5th. He had had a slight chill the day previous. On the morning of the fifth, he attempted to go on with his duties, but was obliged to discontinue the attempt, and, at evening, he took to the bed. I found him with accelerated pulse; skin hot, but bathed in profuse perspiration; headache; thirst. The treatment consisted in the administration of a few grains of Dovers powder.

6th. I did not see him until noon. He had passed a very disturbed night, manifesting pretty constant delirium, wishing to get out of bed, imagining he was engaged at the store, etc. The perspiration subsided shortly after my visit the evening previous, and the skin had been, as it now was, hot and dry. Complained of severe cephalalgia. Pulse 120, and vigorous. Eyes presented an expression of wildness. Constant and great thirst. Tongue lightly coated. Respiration accompanied by frequent sighing. No alvine dejections had occurred since the 4th, on which day he had taken cathartic medicine. No vomiting, nor nausea. I directed ice cap to head, and Proto-Chlor. Hydrarg. grs. xii, to be followed by Seidlitz powders, until free cathartic operations were procured. At evening, the medicine had operated freely, but the symptoms continued without much abatement. I did not perceive, on this day, any delirium, but it was occasionally manifested in my absence. Treatment: warm pediluvia, and ice to head continued.

7th. Night had been passed in constant wakefulness and jactitation, with much active delirium. Other symptoms unchanged. A venesection of ten ounces was practiced, which reduced, for a short time, in a sensible manner, the force and frequency of the pulse, and the heat of skin. At evening, however, the skin having regained its heat, and the pulse its volume and vigor, a second venesection, of from twelve to fourteen ounces, was practiced. The pulse and skin were sensibly affected by this bleeding, and indications of faintness occurred, notwithstanding the patient was recumbent. The prescription for the night was, Pulv. Doveri. grs. iii, every four hours. Ice to head continued.

8th. Night had been less disturbed, but patient had obtained little or no sleep. Constant delirium, the mind laboring with an unceasing succession of insane ideas. Frequent efforts to get out of bed. The pulse, on this morning, were less frequent, being 100, and less vigorous. Less heat of skin. Makes no complaint, and says he feels pretty well. At noon the febrile movement became increased. Ou this day he had several liquid dejections. He manifested more delirium during the daytime than previously. He was almost unceasingly in motion, frequently attempting to get out of bed; talking incoherently, but generally occupied for an instant with a delirious idea, and the delusions constantly varying. Abdomen somewhat tympanitic. Pressure over the right iliac region caused pains, and gurgling. Treatment; S. morphiæ gr. 1-6. Pulv. Camphoræ grs. ii, every four hours.

9th. Had passed a very restless night. Delirium constant and active. For example, he imagined, at one time, that his watcher was a thief, and with this idea, he sprang from the bed, grasped him by the collar, and called for help so loudly as to awaken, and bring into the chamber, other members of the family who were sleeping in an adjoining apartment. Is constantly wandering to-day, but disposed to sleep. Sleeps rather heavily; frequently with eye-lids partially opened. Pulse from 110 to 120. Tongue inclined to be dry. Skin hot. Sighing respiration has ceased. Discharges from bowels have been frequent, which are liquid, and often escape into the bed without his consciousness, or notice. The condition of the bowels presenting the most urgent symptoms, two enemas of T. opii. ℥i, (each) were administered, which restrained, for a time, the diarrhoea, and this constituted the medical treatment for the day. Dr. Treat visited, at evening, in consultation, and the following treatment was agreed upon:—S. Morphiæ gr. 1-6, Pulv. Camphoræ grs. ii, every four hours. Enemas of T. Opii ℥i, every six hours, if alvine discharges continue to be frequent.

10th. Passed as restless a night as heretofore, sleeping very seldom, and but a few moments at a time. Delirium constant, and active. On one occasion he had hysterical emotions—weeping long and loudly, under the idea that he had committed some henious fault. A similar paroxysm occurred during the day. The bowels moved but twice, so that no enemas were administered. To-day, up to 4 P. M., he continued as actively delirious as through the night. His efforts to get out of bed rendered it necessary to watch him constantly. Morphiæ gr. 1-6, and P. Camphor. grs. ii, were given at irregular intervals, more frequently than during the night, and appeared, for a short time after each dose, to produce some quiet. Pulse 120. Eyes had a staring expression. There was not, nor had there been previously, increased sensibility to light, and noise. Pupils neither contracted nor dilated, and had free mobility. Tongue coated and dry. Sordes collects on teeth. Several dejections, but not so frequent and profuse as on the day previous. At 2 P. M., Drs. A. S. Sprague, and W. Treat visited in consultation. It was agreed to apply blister to nuchæ, and to exhibit S. Morphiæ in doses of gr. ¼, with P. Camphoræ grs. iii. Shortly after taking the first dose he fell asleep, and slept profoundly for two and a half hours. At the expiration of that time he was roused with some difficulty. He then began to perspire, and sweat profusely for some time. He continued to sleep profoundly, but was without difficulty roused. Skin, at evening, became again hot and dry. Pulse 116. Involuntary dejections frequent and profuse. The directions for the night were to give no medicine unless he became again wakeful and delirious. If this be the case, and the dejections continue, give enema of T. opii ℨi, and, after waiting an hour, give S. Morphiæ gr. 1-4, if the enema does not produce quiet. Give the Morphia without the enema, if dejections do not continue, and wakefulness with delirium recurs. Continue cold applications to head, as heretofore, with sponging of the body, which has heretofore been practiced several times daily. Milk porridge for diet.

11th. Passed a more quiet night. Delirium continues, but less active. Less restlessness. Two doses of S. Morphiæ (gr. 1-4) were given during the night. Dejections not occurring frequently, no enemas were administered. On this day delirium more closely resembled that generally met with in Typhoid fever. Disposed to somnolency. Pulse 116 to 120. Skin sometimes cool, and sometimes hot. Tongue moist, and thickly coated. Involuntary dejections frequent. Blister vesicated well. Treatment consisted of enemas of T. Opii. Essence of beef and milk porridge for diet.

12th. Drs. Sprague and Treat visited in consultation last evening, and it was agreed to give P. Opii gr. i, Proto. Chlor. Hydrarg. grs. ii, every two hours, and laudanum enemas *pro re nata*. Pulse last evening 120. He passed an exceedingly restless night, with constant delirium, and so continues to day. Continues to make efforts to get out of bed. Skin has been cool; no perspiration. Pulse 128, with less force. Has some trouble in deglutition, and articulates with difficulty. The opium has appeared to produce little or no effect. Involuntary dejections continue to be frequent and profuse. Enemas no longer retained. Possesses his strength so as to be able to raise himself quickly, and would get out of bed if not prevented. In the afternoon morphia was substituted for the opium.

On the evening of this day, Drs. S. and T. again visited in consultation. He was then quiet and sleeping. It was agreed to continue the Morphia in doses sufficient to produce quiet. He continued to sleep uninterruptedly for several hours, and, then, on being roused, appeared to have a slight convulsion. On rousing him fully, so that he would reply to questions, he said, in answer to my question how he felt, "first rate." He was kept under the influence of morphia through the night, but whenever the effects passed away, or he was roused, delirious activity returned. Death occurred at 7 A. M., 13th inst. A post mortem examination could not be obtained.

*Remarks.*—The first inquiry of interest and importance relates to the diagnosis. From the prominence of the cerebral symptoms, the question may arise, and did arise in the minds of the reporter, and those associated in consultation, whether the affection was not primitively and essentially encephalitis. The following considerations seem to establish the conclusion that it was a case of fever, of which the cerebral affection, whatever it may have been, was but a complication:—

Acute Encephatitis scarcely ever occurs in this climate, except as a consequence of injury, or, in some very rare instances, of the excessive abuse of alcoholic stimulants.

The development of the disease was like fever, the patient not having been well for several weeks, and several days being occupied in its forming stage.

The cerebral symptoms were gradually developed. There was never that excessive cephalalgia which usually attends acute encephalitis, and this symptom was not more prominent than it frequently, or generally is, in cases of continued fever, and subsided after the second day. The delirium was gradually developed. It was manifested, it is true, the first night after the attack, but became, by no means, a prominent symptom, in the daytime, for the two first days. The character of the delirium was unlike that ordinarily occurring in connection with acute encephalitis; and, although more active than is common in continued fever, it presented the peculiar characters belonging to the delirium of fever. There was not furious mania, but the delirium was characterised by constant activity of the mental faculties with a constant succession of delusions. The patient, at all times, exhibited coherency for the instant that his attention was directed by questions to a pasticular subject. His replies to questions were consistent. In short, the delirium was like that of typhus, except it was more active. That exaltation of the senses, which attends encephalitis, was not present, denoted by sensitiveness to light and sounds. Injection of the conjunctiva was not present. Vomiting, which very constantly attends encephalitis, was not present. Costiveness usually attends encephalitis, but in this case, profuse diarrhoea was one of the most prominent and troublesome features. Abdominal tenderness, especially over the lower portion of the ileum, and tympanites, which belong to the history of so large a proportion of the cases of continued fever, were present.

There were no evidences of the products of inflammation, i. e. symptoms of the second stage of encephalitis, in which oppression of the intellect is occasioned by the effusion of fibrin and serum beneath the aracknoid. On the contrary, the cerebral derangement indicated excitation up to the close of life, the pulse, too, preserving its frequency, etc.

There was not that tolerance of blood-letting which obtains in encephalitis more than in any other local inflammation. Moderate bleeding, in the recumbent posture, produced marked effects upon the circulation. The blood, which was received into a wash bowl, did not present any evidence of an excess of fibrin.

On a reviewal of the foregoing facts, and by instituting comparison with the diagnostic symptoms of Fever on the one hand, and Encephalitis on the other, the evidence, positive and negative, for the former, must be sufficiently apparent. The practical importance of the discrimination is obvious Admitting that encephalic inflammation existed, it is not merely a matter of scientific interest to distinguish between a primitive local inflammation, accompanied, of course, by symptomatic fever, and an essential fever, embracing a local inflammation as a complication, but the treatment will be materially affected by the distinction. An inflammation *per se*, demands a vigorous prosecution of those measures which are calculated to abate inflammatory action, but the same vigor of practice might be highly injudicious when the same inflammation occurs as a complication of fever, since, if the inflammatory action be subdued, the patient has still to pass through the febrile career, and the means adapted to the former may compromise the power requisite for the latter. The diagnosis is not in all instances easy, especially in children, and in adults if the history of the case be imperfectly known from its commencement. Cruveilhier frankly confesses that he has often erred in confounding fever with encephalitis.

Viewing, then, the foregoing case as one of fever, the question may arise, what was the pathological condition of the encephalon. This question, it is true, is to be answered in a certain sense speculatively, but we have always to decide speculatively, in the same sense, on the pathological conditions of internal organs while the patient lives. The ordinary delirium of fever, it is sufficiently ascertained, does not necessarily involve notable inflammation of the encephalic structures. Its active character in this instance, however, may lead to the supposition that inflammation existed. But acute inflammation sufficient to produce cerebral symptoms of so prominent a character, could hardly be expected to continue for the space of eight days, without giving rise to the usual products of inflammatory action, to wit, effusion of lymph and serum; and the presence of these should have produced a marked change in the cerebral and other symptoms. Taking this into consideration, in connection with the absence of those symptoms so generally associated with acute inflammation of the encephalic structures already mentioned, and the presumption is, that, had an autopsy been obtained, the evidences of inflammation would have been found to have consisted in more or less vascular injection, and opacity of the aracknnoid. In view of the abdominal symptoms, also, we might confidently have expected to find more or less disease of the glands of Peyer.

As a case of fever, this is chiefly remarkable from its gravity, and the great preponderance and active character of the cerebral symptoms, in the latter particular contrasting strongly with the generality of cases occurring in this region.

BUFFALO, Oct., 1848.

